# Advanced glycation endproducts, dityrosine and arginine transporter dysfunction in autism - a source of biomarkers for clinical diagnosis

**DOI:** 10.1186/s13229-017-0183-3

**Published:** 2018-02-19

**Authors:** Attia Anwar, Provvidenza Maria Abruzzo, Sabah Pasha, Kashif Rajpoot, Alessandra Bolotta, Alessandro Ghezzo, Marina Marini, Annio Posar, Paola Visconti, Paul J. Thornalley, Naila Rabbani

**Affiliations:** 1 Warwick Medical School, University of Warwick, Clinical Sciences Research Laboratories, University Hospital, Coventry, UK; 2 Department of Experimental, Diagnostic and Specialty Medicine, School of Medicine, University of Bologna, Via Belmeloro 8, 40126 Bologna, Italy; 3 Department of Computer Science, University of Birmingham, Birmingham, UK; 4 Don Carlo Gnocchi Foundation ONLUS, IRCCS “S. Maria Nascente”, Via Alfonso Capecelatro 66, 20148 Milan, Italy; 5 Child Neurology and Psychiatry Unit, IRCCS Institute of Neurological Sciences, Via Altura, 3, 40139 Bologna, Italy; 6 Department of Biomedical and Neuromotor Sciences, University of Bologna, Via Altura 3, 40139 Bologna, Italy; 7 Zeeman Institute for Systems Biology & Infectious Disease Epidemiology Research, Senate House, University of Warwick, Coventry, CV4 7AL UK; 8 Research Technology Platform–Proteomics, University of Warwick, Coventry, UK

**Keywords:** Autism spectrum disorder (ASD), Advanced glycation endproducts (AGEs), Oxidative stress, Amino acid metabolome, Machine learning

## Abstract

**Background:**

Clinical chemistry tests for autism spectrum disorder (ASD) are currently unavailable. The aim of this study was to explore the diagnostic utility of proteotoxic biomarkers in plasma and urine, plasma protein glycation, oxidation, and nitration adducts, and related glycated, oxidized, and nitrated amino acids (free adducts), for the clinical diagnosis of ASD.

**Methods:**

Thirty-eight children with ASD (29 male, 9 female; age 7.6 ± 2.0 years) and 31 age-matched healthy controls (23 males, 8 females; 8.6 ± 2.0 years) were recruited for this study. Plasma protein glycation, oxidation, and nitration adducts and amino acid metabolome in plasma and urine were determined by stable isotopic dilution analysis liquid chromatography-tandem mass spectrometry. Machine learning methods were then employed to explore and optimize combinations of analyte data for ASD diagnosis.

**Results:**

We found that children with ASD had increased advanced glycation endproducts (AGEs), *N*_ε_-carboxymethyl-lysine (CML) and *N*_ω_-carboxymethylarginine (CMA), and increased oxidation damage marker, dityrosine (DT), in plasma protein, with respect to healthy controls. We also found that children with ASD had increased CMA free adduct in plasma ultrafiltrate and increased urinary excretion of oxidation free adducts, alpha-aminoadipic semialdehyde and glutamic semialdehyde. From study of renal handling of amino acids, we found that children with ASD had decreased renal clearance of arginine and CMA with respect to healthy controls. Algorithms to discriminate between ASD and healthy controls gave strong diagnostic performance with features: plasma protein AGEs—CML, CMA—and 3-deoxyglucosone-derived hydroimidazolone, and oxidative damage marker, DT. The sensitivity, specificity, and receiver operating characteristic area-under-the-curve were 92%, 84%, and 0.94, respectively.

**Conclusions:**

Changes in plasma AGEs were likely indicative of dysfunctional metabolism of dicarbonyl metabolite precursors of AGEs, glyoxal and 3-deoxyglucosone. DT is formed enzymatically by dual oxidase (DUOX); selective increase of DT as an oxidative damage marker implicates increased DUOX activity in ASD possibly linked to impaired gut mucosal immunity. Decreased renal clearance of arginine and CMA in ASD is indicative of increased arginine transporter activity which may be a surrogate marker of disturbance of neuronal availability of amino acids. Data driven combination of these biomarkers perturbed by proteotoxic stress, plasma protein AGEs and DT, gave diagnostic algorithms of high sensitivity and specificity for ASD.

**Electronic supplementary material:**

The online version of this article (10.1186/s13229-017-0183-3) contains supplementary material, which is available to authorized users.

## Background

Autism spectrum disorders (ASD) are defined as developmental disorders mainly affecting social interactions and range of interests and causing a wide spectrum of other disabilities, such as speech disturbances, repetitive and/or compulsive behaviors, hyperactivity, anxiety, and difficulty to adapt to new environments, with or without cognitive impairment [1]. The high heterogeneity of the clinical presentation makes diagnosis of ASD difficult and uncertain, particularly at the early stages of development. Discovery and development of robust biomarkers for diagnosis and progression of severity of ASD is expected to facilitate earlier diagnosis and intervention. It will also likely reveal new causative factors [2, 3]. In particular, alteration in the metabolome and specific damaging biochemical modifications may reveal the presence of a shared metabolic impairment in children with an otherwise highly heterogeneous background, thus shedding some light on the etiopathogenesis of ASD. Genetic causes of ASD are evident in about 30–35% of cases. For the remaining 65–70% of patients, it is generally agreed that ASD results from the combination of environmental factors with multiple de novo mutations, copy number variation, and rare genetic variants, each possibly lending to additive effects. Environmental factors may also be involved and reflected in epigenetic modifications [4]. Transcriptomic, proteomic, and metabolomic profiling have been proposed for diagnosis of ASD, with diagnostic performance judged by area under-the-curve of receiver operating characteristic (AUROC) plot of 0.73–0.91 [5–7]. It is expected that improved diagnostic performance may be achieved with a relatively small number of biomarker analytes linked to the pathogenic mechanism of ASD.

**Fig. 1:**
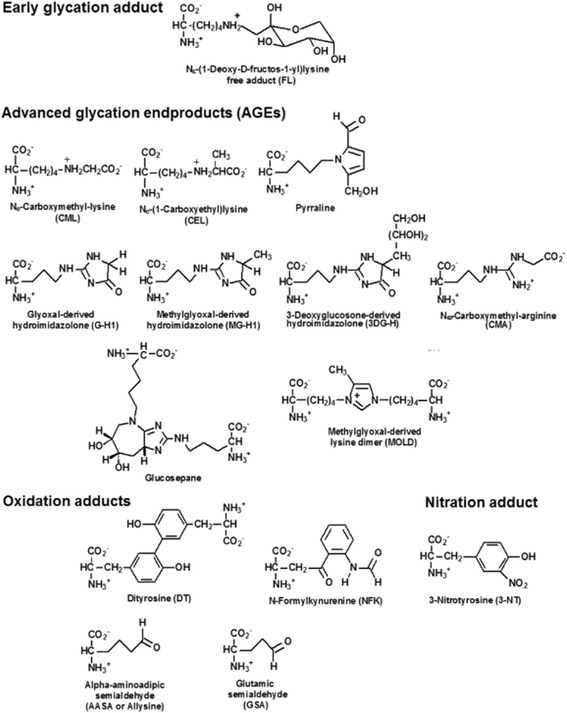
Protein glycation, oxidation, and nitration free adducts. Ionization status at physiological pH is shown. For related adduct residues of proteins, alpha-amino-NH_3_^+^ and terminal carboxylate –CO_2_^−^ groups are moieties of as peptide bonds –NH–CO– with amino acid residues immediately before and after in the peptide backbone

Impairment of protein homeostasis leading to proteotoxic stress and activation of the unfolded protein response (UPR) has been implicated in ASD [8]. Drivers of impaired protein quality are increased spontaneous modifications by glycation, oxidation, and nitration [9]. Glycation of proteins occurs by spontaneous reaction of proteins with glucose, reactive dicarbonyl metabolites, glyoxal, methylglyoxal (MG), and 3-deoxyglucosone (3-DG), and other saccharides and saccharide derivatives. Protein glycation adducts are classified as early stage glycation adducts—such as *N*_ε_-fructosyl-lysine (FL) residues formed by glycation of proteins by glucose—and late-stage adducts, advanced glycation endproducts (AGEs)—such as *N*_ε_-carboxymethyl-lysine (CML) and glucosepane (GSP) residues—formed by the degradation of FL residues, hydroimidazolones G-H1, MG-H1, and 3DG-H formed by the modification of arginine residues by glyoxal, MG and 3-DG, respectively, *N*_ω_-carboxymethylarginine (CMA)—also formed by the reaction of glyoxal with arginine residues, and methylglyoxal-derived lysine crosslink (MOLD). Protein oxidation occurs by the reaction of proteins with reactive oxygen species (ROS) and is increased in oxidative stress. Examples of protein oxidation adducts are dityrosine (DT), *N*-formylkynurenine (NFK), α-aminoadipic semialdehyde (AASA), and glutamic semialdehyde (GSA) residues. Oxidative stress has been implicated as a contributory factor in the development of ASD [10–12]. Increased oxidative damage associated with oxidative stress and neuroinflammation may be common features of ASD in children. Protein nitration occurs by the reaction of proteins with reactive nitrogen species such as peroxynitrite. The main adduct formed by protein nitration is 3-nitrotyrosine (3-NT) residues (Fig. 1). Increased protein damage by these mechanisms may lead to activation of the UPR to counter the proteotoxic threat and related inflammatory response [13, 14].

Glycated, oxidized, and nitrated proteins undergo proteolysis to form related glycated, oxidized, and nitrated amino acids—also called glycation, oxidation, and nitration free adducts. Glycated, oxidized, and nitrated amino acids are released into plasma and are excreted in urine. Urinary excretion of glycation, oxidation, and nitration free adducts are approximate measures of whole body fluxes of protein glycation, oxidation, and nitration, respectively. There are also minor contributions to the pool of these metabolites by direct glycation, oxidation, and nitration of amino acids and absorption from food after digestion of damaged proteins therein [9]. Insight into renal handling of amino acids by the kidney is gained by deducing the renal clearance (CL) of amino acids from plasma to urine. For low molecular weight metabolites such as amino acids, CL is mainly influenced by renal tubule reuptake of amino acids mediated by amino acid membrane transporters. ASD has been previously associated with homozygous mutations in gene solute carrier family 7, member 5 (SLC7A5) which encodes the large neutral amino acid transporter subunit-1 (hLAT-1); and in males, with rare holomorphic variants of cationic amino acid transporter-3 (CAT-3) mediating uptake of arginine, ornithine, and lysine. These transporters mediate amino acid uptake into the cells, including neurons [15].

In this study, we explore the association of proteotoxic damage with ASD by quantifying levels of protein glycation, oxidation, and nitration adducts in plasma protein and related free adducts in plasma and urine of children with ASD and healthy controls. We also quantify the conventional plasma amino acid metabolome [16] and CL of glycation, oxidation, and nitration free adducts and unmodified amino acids. We then explore the diagnostic potential of these biomarkers by development of diagnostic algorithms with optimum combinations of analyte features. We found evidence of increase of selected plasma protein AGEs and DT in children with ASD and also decreased CL of arginine and CMA. These findings implicate a disturbance of metabolism of dicarbonyl precursors of AGEs and activation of dual oxidase (DUOX) in ASD. The initial evidence given herein suggests combination of plasma protein AGE and DT levels may provide a blood-based test for diagnosis of ASD. Decreased CL of arginine and CMA is proposed to be linked to amino acid transporter dysfunction in ASD, building on increasing evidence of neuronal amino acid availability as a driver in ASD development.

## Methods

### Subject recruitment

**Fig. 2:**
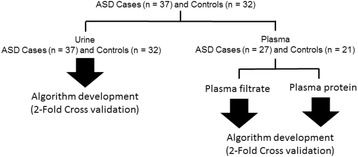
Training and validation subject groups of diagnostic algorithms for detection of autistic spectrum disorder

A total of 69 children were recruited. Of these, 38 had a diagnosis of ASD (29 males and 9 females) and 31 were classified as typically developing (TD) children (23 males and 8 females)—Fig. 2. The age of the two subject groups was not significantly different. Subject age was as follows: ASD group, 7.6 years ±2.0 years, range 5–12 years and TD group, 8.6 ± 2.0 years, range 5–12 years. All ASD subjects received a diagnosis of ASD by two child development experts at the Child Neurology and Psychiatry Unit of the Bellaria Hospital of Bologna (IRCCS Institute of Neurological Sciences), according to the Diagnostic and Statistical Manual of Mental Disorders V (DSM 5 [1] criteria, Autism Diagnostic Observation Schedule (ADOS) [10], Childhood Autism Rating Scale (CARS) [17] and characteristics of onset pattern of ASD defined according to Ozonoff et al. [18]. Developmental and cognitive levels were assessed by Psychoeducational Profile-3 (PEP-3) [19] and Leiter International Performance Scale–Revised (Leiter-R) [20]. For both ASD and TD subjects, exclusion criteria were presence of inflammatory or infective disease and taking antioxidant supplements at the time of study. No subject underwent any surgery intervention in the 4 months prior to blood and urine collection. None of the ASD subjects had active epilepsy at the time of blood and urine sampling. Subjects with ascertained medical and neurological comorbidity were excluded, through a medical work up including electroencephalography (recorded during awake and sleep), cerebral magnetic resonance imaging, standard clinical and neurological examination, neurometabolic, and genetic investigations (including comparative genomic hybridization array, molecular assay for Fragile X and MECP2). Subjects recruited for this study were not taking any medication. TD children were recruited in the local community, with no sign of cognitive, learning, and psychiatric involvement. They were attending mainstream school and had not been subjected to stressful events. Dietary habits were assessed by a Food Questionnaire, built according to the guidelines issued by the Emilia-Romagna Health Authority. No ASD child was on a diet free of gluten or casein. Both patients and controls were on a typical Mediterranean diet, as defined by the prevalence of both simple and complex carbohydrates, use of olive oil, and plenty of fruit [21]. The consumption of vegetables was less than desirable in both patients and controls, although vegetable intake was more limited in ASD patients. Demographic and clinical features of ASD are summarized in Table 1. All subjects were recruited at the Child Neurology and Psychiatry Unit of the Bellaria Hospital of Bologna, Bologna, Italy.

**Table 1:** Demographic and clinical features of the autistic children group

No	Age (years)	Gender	ADOS score	CARS total score	CARS activity level item score (hyperactivity)	CARS body use item score (stereotypes)	CARS total number of items with score ≥ 3	Autism severity level	Cognitive/developmental impairment	Onset pattern (1, early; 2, regressive; 3, mixed)†	Probability of autism‡ from algorithm no.			
											1	2	3	4
1	6.0	m	22	41.0	2.0	3.0	9	Severe	Severe	1	0.93	0.79	0.82	0.80
2	5.6	m	14	34.0	2.0	2.0	2	Mild	Mild	1	1.00	0.71	0.99	0.77
3	5.5	m	18	40.5	2.5	3.5	9	Severe	Moderate	1	1.00	0.86	1.00	0.29
4	5.4	f	17	31.5	2.0	2.0	1	Mild	Mild	3	0.88	0.69	0.55	0.27
5	8.5	m	22	36.5	2.0	2.5	6	Mild	Mild	3	0.98	0.64	1.00	0.88
6	8.7	m	19	44.5	3.0	3.0	11	Severe	Moderate	2	1.00	0.69	1.00	0.74
7	6.8	m	19	36.5	2.5	2.0	7	Mild	Normal IQ	1	1.00	0.63	1.00	0.91
8	5.5	f	15	41.5	2.5	2.0	9	Severe	Borderline IQ	1	0.73	0.77	0.72	0.92
9	11.9	m	22	44.5	2.5	3.0	11	Severe	Severe	2	0.20	0.91	0.34	0.45
10	9.2	f	15	47.5	3.5	3.0	13	Severe	Moderate	1	0.84	0.88	0.94	0.36
11	12.0	m	20	39.0	3.0	3.5	8	Severe	Severe	3	0.94	0.68	0.82	0.63
12	6.2	m	22	42.5	3.0	2.5	10	Severe	Severe	1	1.00	0.69	1.00	0.98
13	6.7	m	21	40.5	2.5	3.0	9	Severe	Severe	2	0.83	0.51	0.85	0.56
14	6.6	m	19	37.0	2.5	3.0	7	Severe	Moderate	1	0.83	0.63	0.74	0.97
15	5.5	m	22	40.5	3.0	3.0	9	Severe	Moderate	3	0.68	0.57	0.82	0.98
16	5.7	m	20	41.5	2.5	3.0	11	severe	severe	1	0.98	0.81	0.98	0.81
17	7.8	m	21	46.0	3.5	4.0	11	Severe	Severe	1	0.83	0.35	0.52	0.79
18	5.7	f	20	43.5	2.5	3.0	10	Severe	Normal IQ	3	0.85	0.39	0.58	0.68
19	7.8	m	20	48.5	3.0	4.0	12	Severe	Severe	2	0.95	0.58	0.93	0.57
20	6.8	m	19	42.0	3.0	3.0	9	Severe	Moderate	2	0.67	0.61	0.54	0.77
21	9.6	m	19	39.5	3.0	3.0	7	Severe	Severe	1	0.55	0.44	0.62	0.51
22	6.2	m	19	41.0	2.5	3.0	9	Severe	Severe	3	0.81	0.32	0.78	0.73
23	8.3	m	16	35.0	2.5	2.0	3	Moderate	Moderate	1	0.70	0.35	0.63	0.56
24	7.1	m	22	41.0	2.5	2.0	7	Severe	Moderate	1	0.83	1.00	0.99	1.00
25	6.2	f	17	38.0	2.0	2.5	7	Severe	Severe	1	0.28	0.82	0.15	0.79
26	10.3	f	21	33.5	2.0	2.0	4	Moderate	Normal IQ	3	0.89	0.55	0.82	0.86
27	10.7	m	19	39.0	2.0	2.5	7	Moderate	Normal IQ	1	0.89	0.60	0.97	0.52
28	6.5	m	17	39.0	3.0	2.5	6	Moderate	Normal IQ	3	ND	ND	ND	0.76
29	8.0	m	22	44.0	2.5	3.0	11	Severe	Moderate	1	ND	ND	ND	0.54
30	7.0	m	18	39.5	2.5	3.0	8	Moderate	Normal IQ	1	ND	ND	ND	0.96
31	9.5	m	21	47.0	3.0	3.5	13	Severe	Severe	1	ND	ND	ND	0.29
32	8.6	m	16	41.5	4.0	2.0	10	Severe	Moderate	3	ND	ND	ND	0.33
33	10.9	m	18	35.0	2.0	3.0	3	Mild	Normal IQ	1	ND	ND	ND	0.28
34	5.8	f	18	43.0	2.5	3.0	11	Severe	Moderate	2	ND	ND	ND	0.30
35	5.3	f	17	39.5	2.5	2.5	6	Moderate	Normal IQ	1	ND	ND	ND	0.62
36	5.3	f	17	41.0	2.5	2.5	7	Severe	Normal IQ	1	ND	ND	ND	0.77
37	8.2	m	20	41.0	2.0	3.5	9	Severe	Moderate	1	ND	ND	ND	0.58
38	11.1	m	13	34.0	2.0	1.5	2	Mild	Normal IQ	3	ND	ND	ND	0.87

†Onset pattern was defined according to Ozonoff et al. [18]

‡Probability the subject has autism estimated from diagnostic algorithms derived from experimental biomarker data—see Table 7

### Blood and urine sampling

Blood was withdrawn in the morning from fasting children. Spot urine samples were the first ones in the morning. Blood samples were collected using ethylenediaminetetra-acetic acid (EDTA) as anticoagulant. Plasma and blood cells were separated immediately by centrifugation (2000*g*, 10 min) and plasma samples stored at − 80 °C until analysis and transferred between collaborating laboratories on dry ice.

### Assay of markers of protein glycation, oxidation, and nitration

The content of glycated, oxidized, and nitrated adduct residues in plasma protein was quantified in exhaustive enzymatic digests by stable isotopic dilution analysis liquid chromatography-tandem mass spectrometry (LC-MS/MS), with correction for autohydrolysis of hydrolytic enzymes [22]. The concentrations of related glycated, oxidized, and nitrated amino acids related free adducts (glycated, oxidized, and nitrated amino acids) in plasma and urine were determined similarly in plasma and urine ultrafiltrate, respectively. Ultrafiltrate of plasma (50 μL) was collected by microspin ultrafiltration (10 kDa cut-off) at 4 °C. Retained protein was diluted with water to 500 μL and washed by 4 cycles of concentration to 50 μL and dilution to 500 μL with water over the microspin ultrafilter at 4 °C. The final washed protein (100 μL) was delipidated and hydrolysed enzymatically as described [22, 23]. Ultrafiltrate of urine (50 μL) was collected by microspin ultrafiltration (3 kDa cut-off) at 4 °C.

Protein hydrolysate (25 μL, 32 μg equivalent) or ultrafiltrate (5 μL) was mixed with stable isotopic standard analytes (amounts as given previously [24]) and analyzed by LC-MS/MS using an Acquity™ UPLC system with a Xevo-TQS tandem mass spectrometer (Waters, Manchester, UK). Samples are maintained at 4 °C in the autosampler during batch analysis. The columns were 2.1 × 50 mm and 2.1 mm × 250 mm, 5 μm particle size Hypercarb™ (Thermo Scientific), in series with programmed switching, at 30 °C. Chromatographic retention was necessary to resolve oxidized analytes from their amino acid precursors to avoid interference from partial oxidation of the latter in the electrospray ionization source of the mass spectrometric detector. Analytes were detected by electrospray positive ionization and mass spectrometry multiple reaction monitoring (MRM) mode where analyte detection response was specific for mass/charge ratio of the analyte molecular ion and major fragment ion generated by collision-induced dissociation in the mass spectrometer collision cell. The ionization source and desolvation gas temperatures were 120 and 350 °C, respectively; cone gas and desolvation gas flow rates were 99 and 900 L/h; and the capillary voltage was 0.60 kV. Argon gas (5.0 × 10^−3^ mbar) was in the collision cell. For MRM detection, molecular ion and fragment ion masses and collision energies optimized to ± 0.1 Da and ± 1 eV, respectively, were programmed [22]—Additional file 1: Table S1. Analytes determined were glycation adducts—FL, and AGEs, CML, *N*_ε_-(1-carboxyethyl)lysine (CEL), pyrraline, CMA, G-H1, MG-H1, 3DG-H, MOLD and GSP; oxidation adducts—DT, NFK, AASA, GSA; nitration adduct, 3-NT; and all major amino acids. Oxidation, nitration, and glycation adduct residues are normalized to their amino acid residue precursors and given as millimoles/mole amino acid modified, and related free adducts are given in nanomolar. Chemical structures and biochemical and clinical significance of these analytes have been described elsewhere [9, 25]. Renal clearance (CL) of glycation, oxidation, and nitration free adducts and unmodified amino acids was deduced from plasma and spot urine collections: CL (μL/mg creatinine or mL/mg creatinine) = [analyte]_Urine_ (nmol/mg creatinine)/[analyte]_Plasma_ (pmol/mL or nmol/mL).

### Machine learning analysis

The objective was to distinguish between children with ASD and healthy controls. In all cases, the diagnostic algorithms were trained on 50% of the cases and controls (training subset) before being used to predict the disease class for each sample in the remaining subjects (test set), twofold cross-validation. The outcome was to assign, for each test set sample, a set of probabilities corresponding to each of the ASD/control groups—the group assignment being that for which the probability is highest. Test data were held separate from algorithm training; algorithm settings were not adjusted once we began to analyze the test set data—thereby guarding against overfitting and hence providing a rigorous estimate of predictive performance. Four algorithm types were tested for performance: random forests, logistic regression, ensemble classifier, and support vector machines (SVMs) [26–28]. During the algorithm training, we used the complete panel of protein glycation, oxidation, and nitration adducts as features and developed algorithms for each analyte type: plasma protein adduct residues, plasma free adducts, and urinary free adducts. For the latter two, unmodified amino acids were also included as features. The aim during the training was to select the set of features that accomplishes the highest performance. The machine learning experiments initially explored using all metabolite features. Subsequent selection of a subset of discriminant biomarker features improved the algorithm performance. For the biomarker selection, we used a sequential feature selection approach. The biomarker feature selection and classifier selection were made on the basis of algorithm performance defined by classification accuracy, sensitivity, specificity, area under-the-curve of the receiver operating characteristic curve (AUROC), positive likelihood ratio, negative likelihood ratio, positive predictive value, negative predictive value, and *F*-measure. For each performance metric, the mean and 95% CI was determined and reported. The algorithm training and testing was repeated 10 times, without altering the algorithm parameters, with 50% data split, to test for algorithm’s robustness against any bias towards data split. We developed our computer programs using Statistics and Machine Learning Toolbox of MATLAB® (MathWorks, Inc., Natick, USA), with a linear kernel SVM and sequential minimal optimization (SMO).

### Statistical analysis

Data are presented as mean ± SD for parametric distributions and median (lower–upper quartile) for non-parametric distributions. The test for normality of data distribution applied was the Kolmogorov–Smirnov test. Significance was evaluated by Student’s *t* test or by Mann–Whitney *U* test for parametrically or non-parametrically distributed data, respectively. Bonferroni correction was made for analysis of multiple analytes without preconceived hypothesis. Correlation analysis was performed by Spearman’s rho method with continuous variables. For clinical categorical variables with ≥ 6 categories, Spearman correlation was performed—assuming approximation to a continuous variable [29]; for other categorical variables, significance of difference of biomarker data distributions between categories was assessed by one-way ANOVA for parametric data and Kruskal—Wallis *H* test. Data were analyzed using SPSS, version 24.0.

For power analysis in the study design, we chose the level of the irreversible oxidative damage marker DT in plasma protein. In healthy human subjects, plasma protein DT was 0.0287 ± 0.0027 mmol/mol tyr (*n* = 29) in previous studies [30]. We designed our study to detect a 50% increase in plasma protein DT at the 0.01% significance level, for which ≥ 18 case and control samples were required. Post hoc analysis revealed an 88% increase with *P* = 0.00017, after Bonferroni correction of 14, 15 or 20 as appropriate with 27 cases and 21 controls, suggesting the study was adequately powered for this key target analyte.

## Results

### Children with autistic spectrum disorder recruited for this study

Thirty-eight children with ASD were recruited for this study. The distribution of severity of ASD in this subject group recruited was (number of cases) mild (6), moderate (6), and severe (26). The distribution of cognitive/developmental impairment was (number of cases) normal/borderline IQ (11), mild (3), moderate (12), and severe (12). The distribution of onset pattern of ASD was (number of cases) early (22), regressive (6), and mixed (10). The ADOS score ranged from 13 to 22 and the CARS total score from 31.5 to 48.5.

### Plasma protein glycation, oxidation, and nitration

In plasma protein, protein content of AGEs—CML, MG-H1 and CMA—were increased in children with ASD, with respect to healthy controls, whereas plasma protein content of AGE, 3DG-H, was decreased in children with ASD, with respect to healthy controls. Plasma protein content of the oxidative damage adduct, DT, was increased in children with ASD, with respect to healthy controls. Only changes in CML, CMA, and DT remained significant after Bonferroni correction for measurement of multiple analytes (Table 2). In correlation analysis, highly significant positive correlations (*P* < 0.01, Spearman) were of CML with DT, G-H1 with MG-H1 and DT, MG-H1 with CMA, CMA with DT, and AASA with GSA—Additional file 1: Table S2. No correlation or association of glycation, oxidation, and nitration adduct residues was found with demographic and clinical features. There was no significant difference of these variables between subject groups of different genders with and without ASD.

**Table 2:** Glycation, oxidation, and nitration adduct residue content of plasma protein

Glycation markers	Healthy controls	ASD	*P* value
FL (mmol/mol lys)	1.27 ± 0.39	1.41 ± 0.53	NS
CML (mmol/mol lys)	0.158 ± 0.026	0.190 ± 0.038	0.0018*
CEL (mmol/mol lys)	0.117 ± 0.044	0.092 ± 0.054	NS
G-H1 (mmol/mol arg)	0.012 ± 0.005	0.016 ± 0.011	NS
MG-H1 (mmol/mol arg)	0.473 ± 0.074	0.535 ± 0.100	0.021
3DG-H (mmol/mol arg)	0.165 ± 0.037	0.138 ± 0.027	0.0052
CMA (mmol/mol arg)	0.054 (0.043–0.067)	0.077 (0.066–0.101)	0.000082**
MOLD (mmol/mol lys)	0.027 ± 0.011	0.025 ± 0.016	NS
GSP (mmol/mol lys)	0.514 ± 0.111	0.571 ± 0.206	NS
DT (mmol/mol tyr)	0.025 (0.019–0.031)	0.047 (0.035–0.094)	0.000012***
NFK (mmol/mol trp)	15.6 ± 1.7	15.0 ± 1.5	NS
AASA (mmol/mol lys)	0.154 ± 0.048	0.152 ± 0.081	NS
GSA (mmol/mol arg)	0.639 ± 0.327	0.713 ± 0.350	NS
3-NT (mmol/mol tyr)	0.0056 (0.0045–0.0069)	0.0053 (0.0045–0.0064)	NS

Data are median (lower–upper quartile); healthy controls, *n* = 21, and ASD, *n* = 27. Significance (Mann–Whitney *U*)

*P < 0.05, **P < 0.01, and ***P < 0.001 after Bonferroni correction of 14 applied

### Plasma glycated, oxidized, and nitrated amino acids and amino acid metabolome

For glycated, oxidized, and nitrated amino acid concentration in plasma, FL, G-H1, and NFK were decreased whereas CMA, AASA, and GSA were increased in children with ASD, with respect to healthy controls. Only increase in CMA remained significant after Bonferroni correction (Table 3). In correlation analysis, highly significant positive correlations were of pyrraline with MG-H1 and 3DG-H, FL with CML, G-H1 and MG-H1, CEL with MG-H1 and CMA, MG-H1 with 3DG-H, and CMA with AASA. There were highly significant negative correlations of pyrraline with NFK, CMA with MOLD, and MOLD with AASA—Additional file 1: Table S3.

**Table 3:** Plasma and urinary glycation, oxidation, and nitration free adduct in plasma filtrate

Amino acid	Plasma (nM)			Urine (nmol/mg creatinine)		
	Healthy controls	ASD	*P* value	Healthy controls	ASD	*P* value
FL	1489 (987–1863)	751 (361–1.570)	0.047	56.7 (33.8–128.7)	90.8 (42.4–167.9)	
CML	807 (587–1051)	853 (219–1222)		26.2 (19.7–34.7)	33.1 (26.6–42.6)	0.016
CEL	402 (298–477)	420 (310–599)		0.435 (0.202–0.848)	0.472 (0.180–0.940)	
G-H1	0.819 (0.553–1.26)	0.527 (0.366–0.959)	0.037	1.88 (1.11–3.04)	2.57 (1.53–3.75)	0.024
MG-H1	271 (176–475)	335 (213–500)		18.6 (8.00–27.4)	24.5 (9.14–37.6)	
3DG-H	413 (303–637)	360 (280–434)		2.06 (0.587–4.38)	2.92 (1.09–6.26)	
CMA	9.18 (6.67–12.5)	17.7 (13.2–24.8)	0.00052**	1.46 (0.636–1.97)	1.78 (1.14–2.91)	0.037
GSP	12.8 (7.4–17.1)	12.9 (9.3–22.0)		1.58 (1.15–1.98)	1.53 (1.20–1.98)	
MOLD	1.79 (0.800–3.42)	1.10 (0.503–0.2.21)		0.025 (0.013–0.050)	0.040 (0.017–0.068)	0.027
Pyrraline	22.0 (12.2–30.4)	24.2 (19.4–40.6)		20.6 (14.9–44.2)	34.2 (22.7–72.5)	0.047
DT	0.501 (0.286–0.771)	0.676 (0.500–0.847)		0.070 (0.058–0.085)	0.086 (0.075–0.109)	0.0022*
NFK	15.2 (12.5–18.1)	11.3 (6.23–14.3)	0.030	0.117 (0.084–0.231)	0.179 (0.107–0.238)	0.037
AASA	19.7 (16.9–29.1)	30.6 (21.1–46.4)	0.0063	1.08 (0.805–2.76)	1.80 (1.13–2.89)	0.040
GSA	73.9 (53.2–129)	109 (80.1–203)	0.039	17.3 (13.2–22.7)	34.5 (12.7–48.0)	0.0018*
3-NT	1.10 (0.90–1.26)	1.17 (0.79–1.58)		0.0044 (0.001–0.010)	0.0077 (0.003–0.014)	

Data are median (lower – upper quartile); healthy controls, *n* = 21–31, and ASD, *n* = 27–38. Significance (Mann-Whitney *U*)

*P < 0.05 after Bonferroni correction of 15 applied

For the conventional amino acid metabolome, there were increases in arg, gln, glu, and thr and decrease in trp in children with ASD, with respect to healthy controls. None of these changes remained significant after Bonferroni correction (Table 4). There were many highly significant positive correlations between plasma amino acid concentrations—Additional file 1: Table S4. No correlation or association of glycation, oxidation, and nitration free adducts and amino acids was found with demographic and clinical features. There was no significant difference of these variables between genders.

**Table 4:** Plasma and urinary amino acid metabolome

Amino acid	Plasma (μM)			Urine (nmol/mg creatinine)		
	Healthy controls	ASD	*P* value	Healthy controls	ASD	*P* value
Ala	294 ± 71.5	330 ± 98.3		301 (224–432)	392 (305–533)	0.030
Arg	39.4 ± 10.3	48.4 ± 16.6	0.016	52.1 ± 17.1	64.0 ± 18.0	0.014
Asn	33.2 ± 4.57	34.3 ± 10.6		92.4 (72.1–132)	169 (123–236)	0.0012*
Asp	35.5 ± 7.37	37.0 ± 10.0		142 (112–179)	160 (117–241)	
Cys (total)	41.7 ± 10.4	46.1 ± 15.0		25.3 (20.4–35.9)	28.3 (21.1–43.3)	
Gln	463 ± 38.6	496 ± 61.2	0.024	529 (452–704)	741 (609–876)	0.0048
Glu	1672 ± 324	1999 ± 619	0.034	373 (254–495)	475 (367–690)	0.010
Gly	272 (187–1061)	241 (121–515)		1.12 (1.19–2.78)	2.58 (1.84–3.45)	0.019
His	68.0 ± 8.51	72.8 ± 9.96		2.19 ± 0.88	2.90 ± 1.21	0.027
IIe	50.7 ± 15.3	51.6 ± 12.4		13.0 ± 4.37	18.1 ± 6.50	0.0059
Leu	126 ± 34.4	132 ± 25.6		43.1 ± 13.6	52.0 ± 13.5	0.028
Lys	132 ± 20.3	146 ± 36.7		136 (83.3–264)	171 (117–352)	
Met	23.1 ± 6.41	26.2 ± 5.84		17.9 ± 7.98	23.1 ± 7.20	0.0037
Phe	68.9 ± 11.3	68.8 ± 16.8		101 ± 38.9	110 ± 32.9	
Pro	195 ± 65.4	223 ± 94.7		14.5 ± 6.21	20.7 ± 5.37	0.00073**
Ser	128 ± 23.5	132 ± 18.7		461 (413–621)	677 (519–910)	0.0021*
Thr	116 ± 36.0	141 ± 46.8	0.041	216 (170–265)	283 (221–405)	0.0039
Trp	5.22 ± 1.26	3.92 ± 2.40	0.0055	114 ± 73.6	164 ± 48.6	0.0043
Tyr	84.4 ± 21.3	87.9 ± 26.8		204 (133–255)	240 (173–317)	
Val	125 ± 31.3	120 ± 26.0		25.6 ± 9.52	34.8 ± 10.9	0.00073**

Data are Mean ± SD or median (lower–upper quartile); healthy controls, *n* = 21, and ASD, *n* = 27. Significance: *t* test for parametric data and Mann-Whitney *U* for non-parametric data

*P < 0.05 and **P < 0.01 after Bonferroni correction of 20 applied

### Urinary glycated, oxidized, and nitrated amino acids and amino acid metabolome and renal clearance

For the urinary flux of glycated, oxidized, and nitrated amino acids, children with ASD showed increased urinary excretion of CML, G-H1, CMA, MOLD, pyrraline, DT, NFK, AASA, and GSA. Only urinary excretions of DT and GSA remained significant after Bonferroni correction (Table 3). For the urinary flux of unmodified amino acids, children with ASD showed increased urinary excretion of all amino acids except asp, cys, lys, phe, and tyr. Only increases in urinary excretion of asn, pro, ser, and val remained significant after Bonferroni correction (Table 4). There were several highly significant positive correlations between urinary excretions of glycation, oxidation, and nitration adducts and amino acids—see Additional file 1: Table S5 and Table S6.

Renal clearance of CMA, GSP, DT, arg, glu, leu, phe, and thr were decreased and renal clearance of NFK and trp were increased in children with ASD, with respect to healthy controls. Only decreases in renal clearance of arg and CMA remained significant after Bonferroni correction: CL_arg_ decreased 32% and CL_CMA_ decreased 50% in children with ASD, compared to healthy control; *P* < 0.001 (Tables 5 and 6). No correlation or association of these glycation, oxidation, and nitration free adduct and amino acid variables was found with demographic and clinical features. There was no significant difference of these variables between genders.

**Table 5:** Renal clearance of glycation, oxidation, and nitration free adducts

Amino acid	Renal clearance (μL/mg creatinine)		
	Healthy controls	ASD	*P* value
FL^**#**^	0.696 (0.375–1.16)	0.866 (0.384–2.31)	
CML	0.297 (0.249–0.387)	0.259 (0.171–0.782)	
CEL	0.0068 (0.003–0.019)	0.0042 (0.002–0.011)	
G-H1	21.2 (13.1–34.7)	23.9 (15.0–57.7)	
MG-H1^**#**^	0.718 (0.496–1.04)	0.628 (0.363–0.912)	
3DG-H	0.067 (0.033–0.163)	0.087 (0.042–0.131)	
CMA	1.57 (0.997–2.08)	0.791 (0.465–1.36)	0.0011*
GSP	0.121 (0.083–0.274)	0.112 (0.067–0.195)	
MOLD	0.214 (0.075–0.487)	0.269 (0.153–0.656)	
Pyrraline	0.81 (0.58–1.17)	1.07 (0.64–1.96)	
DT	0.119 (0.657–2.02)	0.747 (0.545–0.107)	0.0025
NFK	0.062 (0.038–0.109)	0.096 (0.049–0.139)	0.030
GSA	29.9 (11.1–45.8)	17.8 (1.96–26.1)	
3-NT	0.068 (0.034–0.100)	0.042 (0.036–0.129)	

Data are mean ± SD or median (lower–upper quartile); healthy controls, *n* = 21, and ASD, n = 27. Significance: t-test for parametric data and Mann-Whitney *U* for non-parametric data

*P < 0.05, after Bonferroni correction of 14 applied

^**#**^mL/mg creatinine

**Table 6:** Renal clearance of amino acids

Amino acid	Renal clearance (mL/mg creatinine)		
	Healthy controls	ASD	*P* value
Ala	1.03 (0.746–1.712)	1.27 (0.890–1.65)	
Arg	0.011 (0.009–0.015)	0.008 (0.006–0.010)	0.0019*
Asn	2.80 (2.22–4.76)	4.30 (3.05–7.14)	0.0055
Asp	3.81 (3.04–4.96)	4.73 (2.92–7.82)	
Cys (total)	0.646 (0.518–0.812)	0.629 (0.452–1.133)	
Gln	1.18 (0.913–1.53)	1.54 (1.14–1.96)	0.025
Glu	0.210 (0.158–0.304)	0.243 (0.179–0.357	0.049
Gly	0.0050 (0.002–0.009)	0.0067 (0.004–0.029)	0.018
His	0.029 (0.024–0.040)	0.040 (0.027–0.053)	
IIe	0.262 (0.192–0.347)	0.354 (0.256–0.441)	0.0116
Leu	0.358 ± 0.135	0.402 ± 0.105	
Lys	0.011 (0.005–0.018)	0.0071 (0.005–0.011)	
Met	0.0066 ± 0.0015	0.0056 ± 0.0023	
Phe	1.32 (0.959–2.02)	1.65 (1.33–1.95)	
Pro	0.077 ± 0.031	0.104 ± 0.039	0.0138
Ser	3.66 (2.95–4.79)	5.22 (3.99–6.78)	0.0081
Thr	2.023 ± 0.765	2.44 ± 1.05	
Trp	0.209 (0.155–0.242)	0.287 (0.166–0.497)	0.010
Tyr	0.0209 (0.016–0.025)	0.015 (0.012–0.025)	
Val	0.0017 (0.0014–0.0022)	0.0016 (0.0013–0.0026)	

Data are mean ± SD or median (lower–upper quartile); healthy controls, *n* = 21, and ASD, *n* = 27. Significance: *t* test for parametric data and Mann-Whitney *U* for non-parametric data

*P < 0.05, after Bonferroni correction of 20 applied

**Fig. 3:**
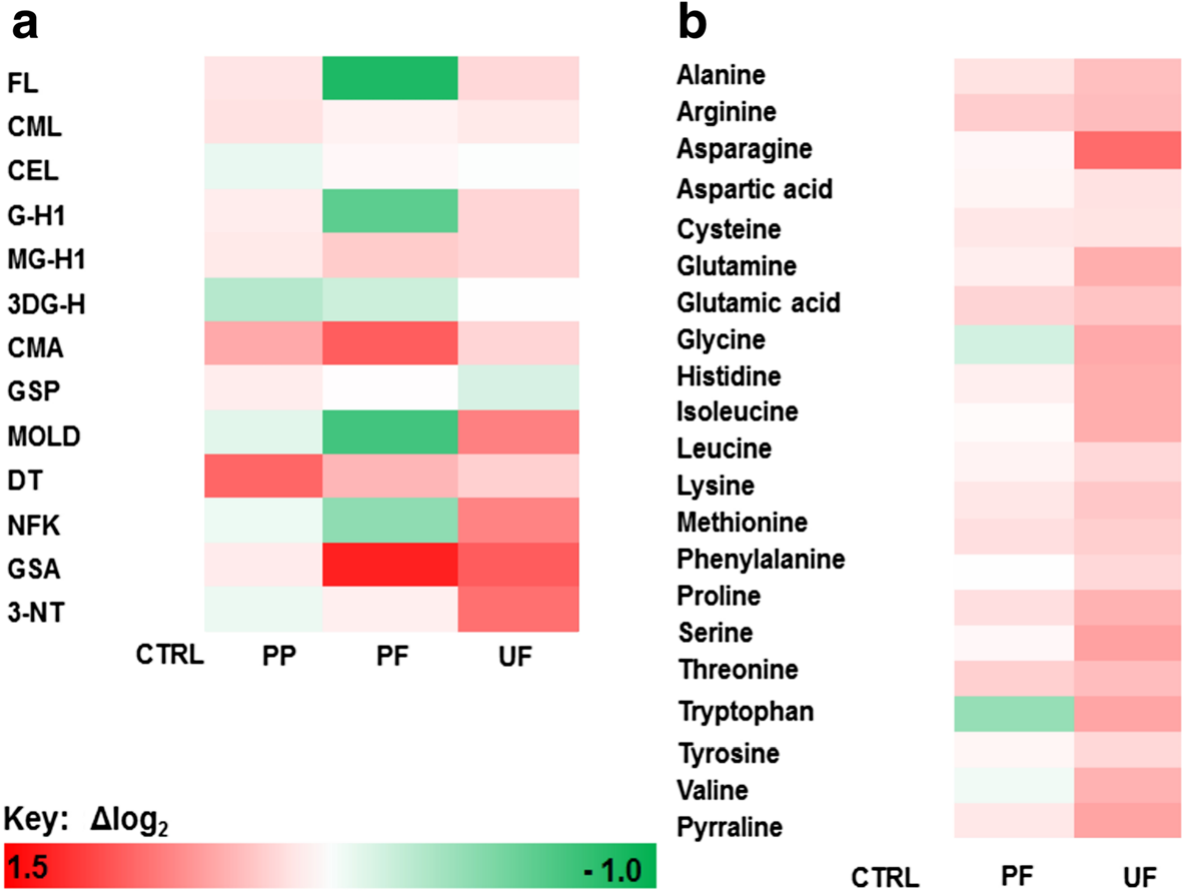
Heat map of changes in glycated, oxidized, and nitrated proteins and amino acids in plasma and urine of subjects with autistic spectrum disorder. **a** Trace level protein glycation, oxidation, and nitration adducts. **b** Amino acid metabolome. Key: CTRL control, PP plasma protein (adduct residues), PF plasma filtrate (free adducts), and UF urine filtrate (free adducts). Data are given in Tables 2, 3, 4, and 5

**Fig. 4:**
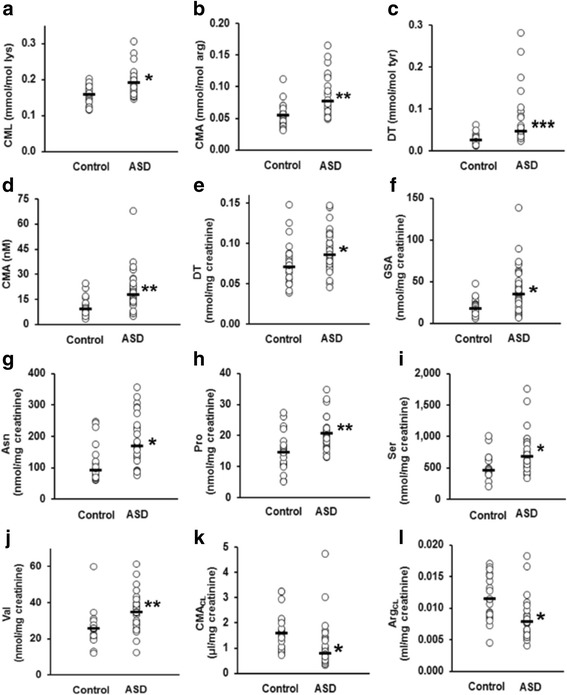
Scatter plots for protein damage biomarker variables changed in children with ASD. Protein adduct residues: **a** CML, **b** CMA, and **c** DT. Plasma free adduct: **d** CMA. Urine free adduct and amino acids: **e** DT, **f** GSA, **g** Asn, **h** Pro, **i** Ser, and **j** Val. Renal clearance: **k** CMA and **l** Arg (an outlier point, 0.0565, was excluded from the ASD data). Significance: One asterisk, two asterisks, and three asterisks indicate *P* < 0.05, *P* < 0.01, and *P* < 0.001, respectively

Changes of glycation, oxidation, and nitration adducts and amino acid metabolome in plasma and urine are summarized in heat maps (Fig. 3a, b). Data distributions of biomarker with significantly different change in the ASD study group after Bonferroni correction are given in Fig. 4.

### Development of diagnostic algorithms for ASD

To explore diagnostic utility of protein glycation, oxidation, and nitration measurements for ASD, we analyzed plasma and urinary amino acid analyte data by a machine learning approach. SVMs was the best-performing method out of the four algorithms that were investigated. Algorithm optimized from twofold cross-validation were as below.

(i) Algorithm-1, developed from plasma protein glycation, oxidation, and nitration adduct residue analytes.

It has the following features: CML, 3DG-H, CMA, and DT. Classification accuracy was 88%, sensitivity 92%, specificity 84%, and AUROC 0.94. A random outcome is 0.50.

(ii) Algorithm-2, developed from plasma glycated, oxidized, and nitrated amino acids and conventional amino acid metabolome.

It has the following features: CML and CMA. Classification accuracy was 75%, sensitivity 81%, and specificity 67% and AUROC 0.80.

(iii) Algorithm-3, developed from plasma protein glycation, oxidation, and nitration adduct residues and plasma glycated, oxidized, and nitrated amino acids and conventional amino acid metabolome combined.

It has the following features: plasma protein CML, 3DG-H, CMA and DT residues, and plasma G-H1 and GSA free adducts. Classification accuracy was 89%, sensitivity 90%, specificity 87%, and AUROC 0.95.

(iv) Algorithm-4, developed from urinary glycated, oxidized, and nitrated amino acids.

**Fig. 5:**
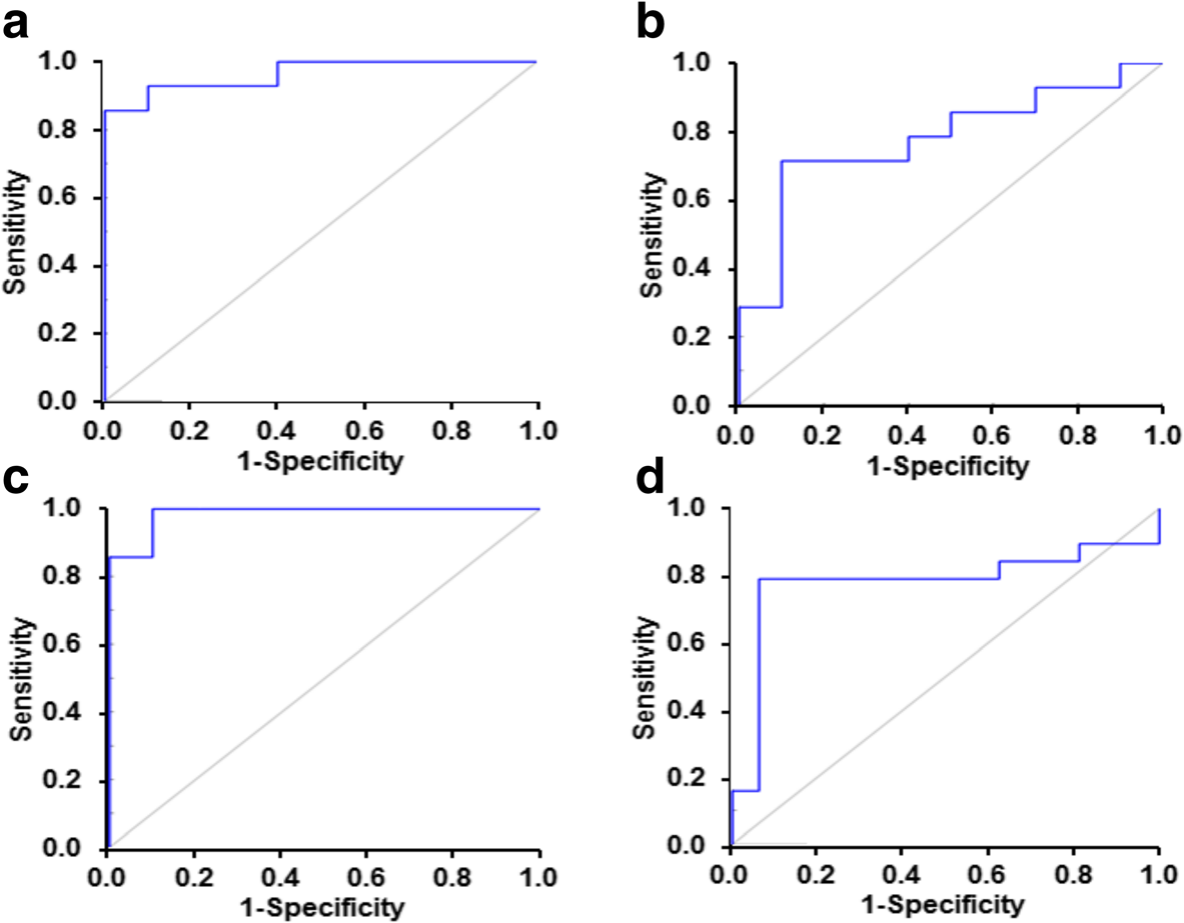
Receiver operating characteristic plots of diagnostic algorithms for detection of autistic spectrum disorder by protein glycation and oxidation adducts. **a** Algorithm-1, plasma protein adduct residues. AUROC = 0.96. **b** Algorithm-2, plasma free adducts. AUROC = 0.78. **c** Algorithm-3, plasma protein adduct residues and free adducts. AUROC = 0.99. **d** Algorithm-4, urine free adducts. AUROC = 0.78. ROC plots are representative results from one run of the classification experiment. A random outcome is AUROC = 0.50

It has the following features: GSA and pyrraline free adducts. Classification accuracy was 77%, sensitivity 77%, specificity 76%, and AUROC 0.79 (Fig. 5, Table 7, and Additional file 1: Table S7).

**Table 7:** Diagnostic algorithms developed for autistic spectrum disorder from plasma and urinary analytes

Algorithm no	1	2	3	4
Compartment and analyte	Plasma protein adduct residues	Plasma amino acids	Plasma protein adduct residues and amino acids	Urinary amino acids
Features	CML, 3DG-H, CMA, and DT	CML and CMA	CML, 3DG-H, CMA, and DT residues with G-H1 and GSA free adducts	GSA and pyrraline free adducts
Accuracy (%)	88.3 (85.5–91.2)	74.8 (71.7–77.9)	89.0 (87.0–91.0)	76.8 (74.6–79.0)
Sensitivity (%)	91.9 (89.1–94.6)	80.5 (75.1–86.0)	90.4 (87.7–93.1)	77.1 (73.4–80.8)
Specificity (%)	83.9 (79.3–88.4)	67.1 (58.9–75.4)	87.3 (84.1–90.5)	76.4 (72.0–80.8)
AUROC	0.94 (0.91–0.96)	0.80 (0.77–0.83)	0.95 (0.94–0.96)	0.79 (0.76–0.81)
Positive likelihood ratio	5.69 (4.49–6.89)	2.85 (2.16–3.55)	7.23 (6.09–8.38)	4.16 (2.88–5.44)
Negative likelihood ratio	0.10 (0.07–0.13)	0.28 (0.21–0.35)	0.11 (0.08–0.14)	0.30 (0.25–0.34)
Positive predictive value (%)	88.2 (85.0–91.4)	77.1 (72.9–81.4)	90.2 (87.9–92.5)	80.6 (77.6–83.5)
Negative predictive value (%)	89.1 (85.5–92.6)	75.0 (70.6–79.4)	88.0 (85.1–91.0)	73.7 (71.0–76.5)
*F* score	0.90 (0.87–0.92)	0.78 (0.75–0.81)	0.90 (0.88–0.92)	0.78 (0.76–0.81)

Algorithm outcomes for twofold cross-validation (10 randomized repeat trials for robustness) using SVMs (95% CI given in brackets)

The diagnostic algorithms were used to deduce the probability of having ASD for each patient diagnosed with ASD by clinical symptoms (Table 1). The association and correlation of these probabilities with clinical features was explored. No significant association or correlation of these probabilities with clinical features (age, ADOS, total CARS, CARS hyperactivity and CARS body use scores, autism severity, cognitive/developmental impairment, and ASD onset pattern) was found.

## Discussion

In this study, we identified changes in plasma protein AGE and oxidation adducts, increased CML, CMA, and DT and decreased 3DG-H in ASD. Combined in an algorithm, these features provided diagnostic performance improved over that previously achieved in transcriptomic, proteomic, and metabolomic studies [5–7]—with AUROC 0.94 and classification efficiency 88%. This was slightly improved by combination with plasma G-H1 and GSA free adducts, with AUROC 0.95 and classification efficiency 89%. This novel biomarker approach focused to protein damage or proteotoxic stress may lead to biochemical-based diagnosis of ASD and suggests that protein AGE and oxidation may be linked to ASD pathogenesis.

**Fig. 6:**
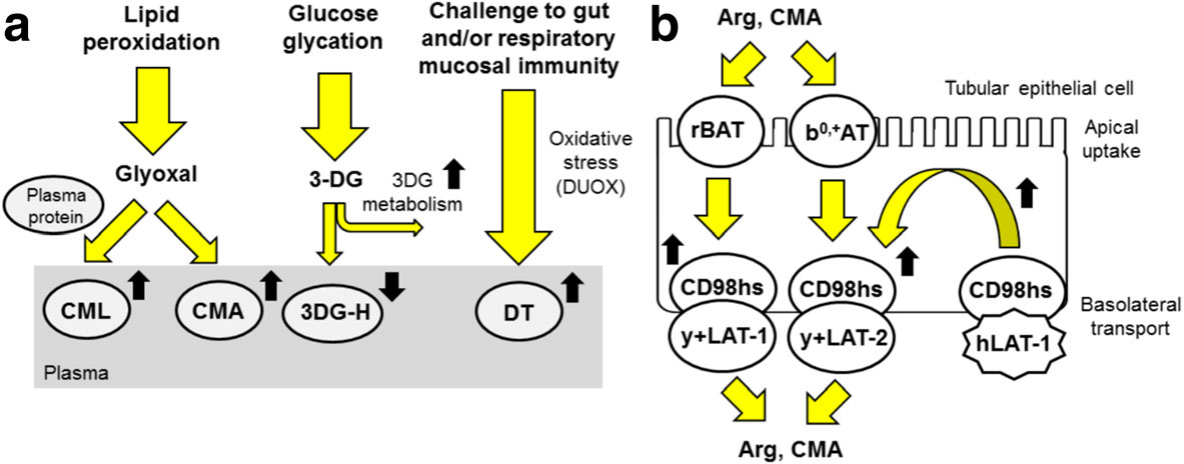
Schematic explanation for changes found in protein damage and amino acids in ASD. **a** Proposed mechanism for observed changes found in plasma protein glycation and oxidation adducts. **b** Transport of arg and CMA across the renal tubular epithelium and proposed mechanism for increased renal CL (increased arg and CMA reuptake). Key: yellow-filled arrows show processes; black-filled arrows show changes observed (**a**) and changes expected (**b**) in ASD

Change in AGE and oxidation adduct content of plasma proteins relates to the rate of protein modification in the plasma compartment and, to a lesser extent, to modifications of plasma proteins in interstitial fluid. The major plasma protein albumin makes repeated cycles from plasma to interstitial fluid and lymph before degradation [31]. CML residues in plasma protein are mainly produced by the oxidative degradation of FL with a usually minor contribution from glycation by glyoxal. CML is also considered to be a marker of oxidative damage [32]. CMA is produced exclusively by the glycation of proteins by glyoxal [9]. Increased formation of glyoxal, mainly sourced from lipid peroxidation, in ASD may explain increases in plasma protein CML and CMA (Fig. 6a). Markers of lipid peroxidation, plasma and red blood cell malondialdehyde, urinary 8-isoprostane-F2a, and hexanoyl-lysine adduct were elevated in ASD [33–35].

DT residue content of plasma proteins was increased in subjects with ASD whereas other oxidative damage markers, NFK, AASA, and GSA, were not. DT residue formation occurs by reaction of tyrosine residues in proteins with ROS and DUOX [36]. The selective increase in DT may suggest a role of increased DUOX activity in subjects with ASD. DUOX expression is increased through activating transcription factor 2 in inflammatory signaling [37]. DUOX has an important role in gut mucosal immunity, host–microbe homeostasis, and signaling for neutrophil recruitment into allergic airways [38, 39]. Gut microbiota may be influential in the development of the behavioral phenotype in ASD children [40] (Fig. 6a).

Decrease of 3DG-H content of plasma protein in subjects with ASD likely reflects decreased concentration of plasma 3-DG. 3-DG is formed by degradation of fructosamine-3-phosphate in the repair of early glycated proteins and degradation of fructose-3-phosphate formed by fructosamine-3-phosphokinase [41, 42] and the slow, non-enzymatic oxidative degradation of glucose and proteins glycated by glucose [43]. It is metabolized to 3-deoxyfructose by aldoketo reductases 1A4, 1B1, and 1B3 which together constitute 3-DG reductase activity [44]. Since FL residue content of subjects with ASD was unchanged, with respect to healthy controls, there is unlikely to be decreased activity of fructosamine-3-phosphokinase in ASD. Rather, increased 3-DG reductase activity may explain decrease in plasma protein 3DG-H residue content in ASD (Fig. 6a).

Combination of markers of oxidative metabolism, DNA oxidation, and methylation in multivariate statistical models was recently found to distinguish between children with ASD and healthy controls [12]. Herein, oxidative damage markers, plasma protein CML and DT and plasma GSA free adduct, emerged as features of diagnostic algorithms for ASD. Measurement of multiple chemically defined markers of protein oxidative damage in plasma and urine compartments in subjects with and without ASD has provided evidence of changes specific to oxidative damage marker type and sample compartment that likely contributed to algorithm development for ASD with improved diagnostic performance.

For unmodified amino acids, we found no changes after correction for multiple measurements in plasma but there were significant increases in urinary excretion of asn, pro, ser, and val. This may relate to impaired tissue uptake and retention of these amino acids in ASD. For modified amino acids, only increased CMA remained significantly increased in plasma for children with ASD. This may relate to proteolysis of plasma proteins and potentially other proteins of increased CMA residue content in ASD. For modified amino acids in urine, urinary excretions of oxidative damage markers, DT and GSA free adducts, were increased in children with ASD after correction for multiple analytes. This may relate to proteolysis of plasma proteins and other proteins with increased oxidative damage and DUOX-catalyzed modification in ASD. The amino acid metabolome in plasma and urine has been explored previously for biomarkers of ASD [7, 45–47]. We confirmed the reported minor increase in plasma arg in ASD, compared to healthy controls, but significance was lost after correction for multiple measurements [48].

Pyrraline is an AGE sourced only from food [49]. Increased urinary pyrraline is indicative of increased food consumption and/or permeability of the gastrointestinal tract to pyrraline. The positive correlation of pyrraline with CML, MG-H1, CMA, DT, and GSA free adducts in urine suggests that increase of these free adducts may be partly due to food consumption.

Exploring changes in renal CL of amino acids provides insight in functional activity of amino acid transporters in the renal tubular epithelium. The deduction of CL herein was an indirect measure based on estimates of urinary analyte/creatinine ratio rather than urinary analyte excretion rate determined in urine collection made over a fixed time interval, usually 24 h. This was done because of difficulties in timed collection of urine in children [50]. Decreased CL relates to increased tubular reuptake and increased amino acid transporter activity. Decreased CL of arg and CMA likely reflects increased reuptake of arg and CMA. Arg uptake by the tubular epithelium is mediated by neutral and basic amino acid transport protein rBAT and solute carrier 7, member 9 (b^0,+^AT) [apical uptake] and CD98 heavy subunit (CD98hs)/y+LAT-2 (solute carrier family 7 member 6) and CD98hs/y+LAT-1 (solute carrier family 7 member 7) complexes [basolateral transport] [51] and this likely mediates renal tubule uptake of arginine derivative CMA too (Fig. 6b). Homozygous mutations of SLC7A5 gene were associated with ASD. SLC7A5 encodes protein hLAT-1 which, together with CD98hs, form the large neutral amino acid transporter involved in maintaining normal levels of brain branched chain amino acids in the brain [52]. Dysfunction of hLAT-1, also found in the renal tubular epithelium in complex with CD98hs [51], may leave the latter more available for complexation with y+LAT-1 and y+LAT-2 and drive increased reuptake and  decreased CL of arginine and CMA. In addition, rare holomorphic variants in males of amino acid transporter CAT-3 were associated with ASD [15]. Disturbance in arginine transporter function may be a common feature of ASD and measure of CL of arginine is an accessible biomarker of this.

Combination of AGE and DT residue content of plasma protein in Algorithm-1 and this combined with G-H1 and GSA free adducts in Algorithm-3 gave the best diagnostic performance for detection of ASD from the analytes determined herein. The absence of conventional, unmodified amino acids from optimum features of the diagnostic algorithms suggests that assay of trace level, AGE and oxidised amino acid residues of plasma protein and free adducts in plasma provides a diagnostic advantage which has not been hitherto explored. It also suggests that AGE and oxidation proteotoxic stress may underly the development of ASD, at least in part. Protein glycation and oxidation adducts from dietary protein contribute to levels of plasma and urinary glycation and oxidation free adducts; whereas glycation and oxidation adduct residues of protein reflect rates of endogenous glycation and oxidation of protein in mainly the vascular compartment. The dominance of plasma protein AGE and oxidation adducts in Algorithm-1 and the modest improvement by addition of plasma G-H1 and GSA free adducts (Algorithm-3) may indicate that there is limited influence of dietary glycated and oxidized proteins to the development of ASD. Rather, challenge to proteostasis by changes in endogenous protein modification by AGEs and DT may contribute to the development of ASD.

## Conclusions

We identified changes in plasma protein glycation and oxidation markers; increased CML, CMA, and DT and decreased 3DG-H that combined in an algorithm gave improved diagnostic performance over other approaches. Increased levels of DT may indicate induction of increased DUOX activity linked to gut mucosa dysfunction. Disturbance of renal handling of arginine and CMA may indicate dysfunctional arginine transporter function common in ASD. Further clinical validation of plasma protein CML, CMA, DT, and 3DG-H may provide improved diagnosis of ASD. For future studies, we suggest firstly validation of the current findings in an independent clinical study group. Thereafter, priorities are investigation of the biomarkers in children younger than 5 years old to assess their ability to improve diagnosis at earlier stages of ASD development, assessment of the biomarkers in prospective studies for prediction of risk of progression to severe symptoms, study of the association of genetic polymorphisms of DUOX and arginine transporters with clinical ASD, preclinical functional genomics of DUOX and arginine transporters with an ASD-like phenotype, and assessment of the specificity of the algorithms for ASD versus other psychiatric conditions.

## Additional file

Additional file 1: Table S1. Mass spectrometric multiple reaction monitoring detection of protein glycation, oxidation and nitration adducts and amino acids. **Table S2.** Correlation analysis – plasma protein glycation, oxidation and nitration adduct residues. **Table S3.** Correlation analysis – plasma protein glycation, oxidation and nitration free adducts. **Table S4.** Correlation analysis – plasma amino acids. **Table S5.** Correlation analysis – urinary protein glycation, oxidation and nitration free adducts. **Table S6.** Correlation analysis – Urinary amino acids. **Table S7.** Confusion matrix of algorithm to identify autistic spectrum disorder. (DOCX 80 kb)

## Abbreviations

3-DG: 3-Deoxyglucosone
3DG-H: Hydroimidazolone AGEs derived from 3-deoxyglucosone
3-NT: 3-Nitrotyrosine
AASA: α-Aminoadipic semialdehyde
ADOS: Autism diagnostic observation schedule
AGE: Advanced glycation endproduct
ASD: Autism spectrum disorder
AUROC: Area under-the-curve of receiver operating characteristic plot
b^0,+^AT: Solute carrier 7, member 9
CARS: Childhood autism rating scale
CAT-3: Cationic amino acid transporter-3
CD98hs: Cluster of differentiation heavy subunit
CEL: *N*_ε_-(1-carboxyethyl)lysine
CL: Renal clearance
CMA: *N*_ω_-carboxymethylarginine
CML: *N*_ε_-carboxymethyl-lysine
DT: Dityrosine
DUOX: Dual oxidase
EDTA: Ethylenediaminetetra-acetic acid
FL: *N*_ε_-fructosyl-lysine
G-H1: Hydroimidazolone AGE derived from glyoxal
GSA: Glutamic semialdehyde
GSP: Glucosepane
hLAT-1: Large neutral amino acid transporter subunit-1
LC-MS/MS: Liquid chromatography-tandem mass spectrometry
Leiter-R: Leiter international performance scale–revised
MG: Methylglyoxal
MG-H1: Hydroimidazolone AGE derived from methylglyoxal MOLD, methylglyoxal-derived lysine crosslink
MRM: Multiple reaction monitoring
NFK: *N*-Formylkynurenine
PEP-3: Psychoeducational profile-3
rBAT: Neutral and basic amino acid transport protein
ROS: Reactive oxygen species
SLC7A5: Solute carrier family 7, member 5
SVM: Support vector machines
TD: Typically developing
UPR: Unfolded protein response
y+LAT-1: Solute carrier family 7 member 7
y+LAT-2: Solute carrier family 7 member 6
