# Supplementary material for: Advanced glycation endproducts, dityrosine and arginine transporter dysfunction in autism - a source of biomarkers for clinical diagnosis
Source: Mol Autism. 2018 Feb 19;9:3. doi: 10.1186/s13229-017-0183-3 (PMC5817812; doi:10.1186/s13229-017-0183-3)
Supplement: Supplementary file 1 — Mass spectrometric multiple reaction monitoring detection of protein glycation, oxidation and nitration adducts and amino acids. Table S2. Correlation analysis – plasma protein glycation, oxidation and nitration adduct residues. Table S3. Correlation analysis – plasma protein glycation, oxidation and nitration free adducts. Table S4. Correlation analysis – plasma amino acids. Table S5. Correlation analysis – urinary protein glycation, oxidation and nitration free adducts. Table S6. Correlation analysis – Urinary amino acids. Table S7. Confusion matrix of algorithm to identify autistic spectrum disorder. (DOCX 80 kb) [file 13229_2017_183_MOESM1_ESM.docx]

**Additonal file**

**Advanced glycation endproducts, dityrosine and arginine transporter dysfunction in autism - a source of biomarkers for clinical diagnosis**

Attia Anwar^1*^, Provvidenza Maria Abruzzo^2,4*^, Sabah Pasha^1^, Kashif Rajpoot^3^, Alessandra Bolotta^2,4^, Alessandro Ghezzo^2^, Marina Marini^2,4^ , Annio Posar^5,6^, Paola Visconti^5^, Paul J Thornalley^1,7^ and Naila Rabbani^1,7,8^

**Table S1.** Mass spectrometric multiple reaction monitoring detection of protein glycation, oxidation and nitration adducts and amino acids.

| Analyte group | Analyte | Rt(min) | Parent ion(Da) | Fragment ion (Da) | CE  (eV) | Neutral fragment loss(es) | Internal standard and amount added |
| --- | --- | --- | --- | --- | --- | --- | --- |
| Glycation | FL | 28.5 | 291.0 | 84.3 | 31 | H_2_CO_2_, fructosylamine | [^2^H_4_]FL, 0.3 pmol |
|  | CML | 28.5 | 204.9 | 130.1 | 12 | NH_2_CH_2_CO_2_H | [^13^C_6_]CML, 0.25 pmol |
|  | CEL | 28.8 | 219.2 | 130.1 | 13 | NH_2_CH(CH_3_)CO_2_H | [^13^C_6_]CEL, 0.25 pmol |
|  | G-H1 | 12.4 | 215.0 | 100.2 | 14 | NH_2_CH(CO_2_H)CH_2_CH=CH_2_ | [^15^N_2_]G-H1, 0.25 pmol |
|  | MG-H1† | 11.6 & 12.5 | 229.2 | 114.3 | 14 | NH_2_CH(CO_2_H)CH_2_CH=CH_2_ | [^15^N_2_]MG-H1, 1.25 pmol |
|  | 3DG-H | 11.2, 12.6 & 13.5 | 319.1 | 114.8 | 20 | NH_2_CH(CO_2_H)CH_2_CH=CH_2_ | [^15^N_2_]3DG-H, 0.25 pmol |
|  | CMA | 12.1 | 233.0 | 70.1 | 27 | H_2_CO_2_, NH_2_C(=NH)NHCH_2_CO_2_H | [^13^C_2_]CMA, 0.25 pmol |
|  | MOLD | 14.0 | 341.2 | 212.3 | 21 | NH_2_CH(CO_2_H)CH_2_CH_2_CH=CH_2_ | [^2^H_8_]MOLD, 0.10 pmol |
|  | GSP | 16.5 | 429.2 | 382.1 | 38 | C_2_H_5_O | [^13^C_6_]Glucosepane, 0.25 pmol |
|  | Pyrraline | 17.9 | 255.2 | 84.3 | 23 | 2-CHO-5-HOCH_2_-pyrrole, H_2_CO_2_ | [^13^C_6_,^15^N_2_]Pyrraline, 1.00 pmol |

**Table S1.** Mass spectrometric multiple reaction monitoring detection of protein glycation, oxidation and nitration adducts and amino acids (cont’d).

| Analyte group | Analyte | Rt (min) | Parent ion  (Da) | Fragment ion (Da) | CE  (eV) | Neutral fragment loss(es) | Internal standard and amount added |
| --- | --- | --- | --- | --- | --- | --- | --- |
| Oxidative | MetSO | 8.7 | 166.1 | 102.2 | 14 | CH_3_-SOH | [^2^H_3_]MetSO, 0.25 pmol |
|  | DT | 19.9 | 361.2 | 315.3 | 15 | H_2_CO_2_ | [^2^H_6_]DT, 0.25 pmol |
|  | NFK | 21.5 | 235.8 | 191.2 | 18 | H_2_CO_2_ | [^15^N_2_]NFK, 0.25 pmol |
|  | AASA | 10.7 | 128.0 | 82.0 | 15 | H_2_CO_2_ | [^2^H_3_]AAA (Rt = 29.4), 2.5 pmol |
|  | GSA | 32.2 | 114.0 | 68.0 | 15 | H_2_CO_2_ | [^2^H_3_]AAA (Rt = 29.4), 2.5 pmol |
| Nitration | 3-NT | 23.2 | 227.1 | 181.2 | 13 | H_2_CO_2_ | [^2^H_3_]3-NT, 0.25 pmol |
| Amino acids | Ala | 5.2 | 90.1 | 44.1 | 8 | H_2_CO_2_ | [^2^H_3_]Ala, 250 pmol |
|  | Arg | 29.2 | 175.2 | 70.3 | 15 | H_2_CO_2_, NH_2_C(=NH)NH_2_ | [^15^N_2_]Arg, 250 pmol |
|  | Asn | 7.2 | 133.2 | 74.1 | 14 | CH_3_CONH_2_ | [^13^C_4_]Asp, 250 pmol |
|  | Asp | 7.5 | 134.1 | 88.0 | 10 | H_2_CO_2_ | [^13^C_2_,^15^N_1_]Asp, 250 pmol |

**Table S1.** Mass spectrometric multiple reaction monitoring detection of protein glycation, oxidation and nitration adducts and amino acids (cont’d).

| Analyte group | Analyte | Rt (min) | Parent ion  (Da) | Fragment ion (Da) | CE  (eV) | Neutral fragment loss(es) | Internal standard and amount added |
| --- | --- | --- | --- | --- | --- | --- | --- |
|  | Cys | 6.6 | 122.0 | 59.0 | 18 | H_2_CO_2_ + NH_3_ | [^13^C_3_,^15^N_1_]Cys, 250 pmol |
|  | Cystine_2_ | 32.6 | 241.1 | 120.1 | 20 | C_3_H_7_NO_2_S (cys) | [^2^H_4_]Cys 250 pmol |
|  | Gln | 9.9 | 147.2 | 84,1 | 16 | H_2_CO_2_ + NH_3_ | [^13^C_5_]Gln, 250 pmol |
|  | Glu | 28.4 | 148.1 | 102.1 | 10 | H_2_CO_2_ | [^13^C_5_]Glu, 250 pmol |
|  | Gly | 4.8 | 76.2 | 30.1 | 6 | H_2_CO_2_ | [^13^C_2_^15^N_1_]Gly, 250 pmol |
|  | His | 32.6 | 156.1 | 93.0 | 22 | H_2_CO_2_ + NH_3_ | [^13^C_6_]His, 250 pmol |
|  | Ile | 31.5 | 132.3 | 86.2 | 10 | H_2_CO_2_ | [^13^C_6_]Ile, 250 pmol |
|  | Leu | 27.6 | 132.3 | 86.2 | 10 | H_2_CO_2_ | [^2^H_3_]Leu, 250 pmol |
|  | Lys | 5.5 | 147.1 | 84.3 | 15 | H_2_CO_2_, NH_3_ | [^13^C_6_]Lys, 250 pmol |
|  | Met | 29.5 | 150.0 | 104.2 | 11 | H_2_CO_2_ | [^2^H_3_]Met, 250 pmol |

**Table S1.** Mass spectrometric multiple reaction monitoring detection of protein glycation, oxidation and nitration adducts and amino acids (cont’d).

| Analyte group | Analyte | Rt (min) | Parent ion  (Da) | Fragment ion (Da) | CE  (eV) | Neutral fragment loss(es) | Internal standard and amount added |
| --- | --- | --- | --- | --- | --- | --- | --- |
|  | Orn | 5.2 | 133.1 | 70.1 | 9 | H_2_CO_2_, NH_3_ | [^2^H_6_]Orn, 50 pmol |
|  | Phe | 17.2 | 166.1 | 103.1 | 26 | H_2_CO_2_, NH_3_ | [ring-^2^H_5_]Phe, 250 pmol |
|  | Pro | 6.6 | 116.1 | 70.1 | 12 | H_2_CO_2_ | [^13^C_5_]Pro, 250 pmol |
|  | Ser | 5.2 | 106.0 | 42.0 | 18 | H_2_CO_2_, H_2_O | [^13^C_3_]Ser, 250 pmol |
|  | Thr | 5.9 | 120.1 | 56.1 | 14 | H_2_CO_2_, H_2_O | [^13^C_4_]Thr, 250 pmol |
|  | Trp | 23.5 | 205.0 | 159.1 | 15 | H_2_CO_2_ | [^15^N_2_]Trp, 250 pmol |
|  | Tyr | 18.3 | 182.1 | 136.2 | 13 | H_2_CO_2_ | [^2^H_4_]Tyr, 250 pmol |
|  | Val | 8.2 | 117.8 | 72.0 | 19 | H_2_CO_2_ | [^2^H_8_]val, 250 pmol |

**Table S2.** Correlation analysis – plasma protein glycation, oxidation and nitration adduct residues.

| Plasma protein glycation and oxidation adduct residues | CML |  |  |  |  |  |  |  |
| --- | --- | --- | --- | --- | --- | --- | --- | --- |
|  | G-H1 |  |  |  |  |  |  |  |
|  | MG-H1 |  | 0.41 |  |  |  |  |  |
|  | CMA |  |  | 0.67 |  |  |  |  |
|  | DT | 0.38 | 0.54 |  | 0.63 |  |  |  |
|  | AASA |  |  |  |  |  |  |  |
|  | GSA |  |  |  |  |  | 0.73 |  |
|  |  | CML | G-H1 | MG-H1 | CMA | DT | AASA | GSA |
|  |  | Plasma protein glycation and oxidation adduct residues | | | | | | |

Correlation coefficients; *Spearman* (P<0.01).

**Table S3.** Correlation analysis – plasma protein glycation, oxidation and nitration free adducts.

| Plasma protein glycation and oxidation free adduct | Pyrraline |  |  |  |  |  |  |  |  |  |  |  |
| --- | --- | --- | --- | --- | --- | --- | --- | --- | --- | --- | --- | --- |
|  | FL |  |  |  |  |  |  |  |  |  |  |  |
|  | CML |  | 0.59 |  |  |  |  |  |  |  |  |  |
|  | CEL |  |  |  |  |  |  |  |  |  |  |  |
|  | G-H1 |  | 0.55 |  |  |  |  |  |  |  |  |  |
|  | MG-H1 | 0.69 | 0.50 |  | 0.44 | 0.44 |  |  |  |  |  |  |
|  | 3DG-H | 0.39 |  |  |  |  | 0.49 |  |  |  |  |  |
|  | CMA |  |  |  | 0.43 |  |  |  |  |  |  |  |
|  | MOLD |  |  |  |  |  |  |  | - 0.48 |  |  |  |
|  | NFK | - 0.41 |  |  |  |  |  |  |  |  |  |  |
|  | AASA |  |  |  |  |  |  |  | 0.42 | - 0.40 |  |  |
|  |  | Pyrraline | FL | CML | CEL | G-H1 | MG-H1 | 3DG-H | CMA | MOLD | NFK | AASA |
|  |  | Plasma protein glycation and oxidation free adduct | | | | | | | | | | |

Correlation coefficients; *Spearman* (P<0.01).

**Table S4.** Correlation analysis – plasma amino acids.

| Plasma amino acid | Arg |  |  |  |  |  |  |  |  |  |  |  |  |  |  |  |  |  |
| --- | --- | --- | --- | --- | --- | --- | --- | --- | --- | --- | --- | --- | --- | --- | --- | --- | --- | --- |
|  | Ala | 0.61 |  |  |  |  |  |  |  |  |  |  |  |  |  |  |  |  |
|  | Asn |  | 0.51 |  |  |  |  |  |  |  |  |  |  |  |  |  |  |  |
|  | Asp | - 0.56 |  |  |  |  |  |  |  |  |  |  |  |  |  |  |  |  |
|  | Cys | 0.40 |  |  |  |  |  |  |  |  |  |  |  |  |  |  |  |  |
|  | Gln |  | 0.46 |  |  |  |  |  |  |  |  |  |  |  |  |  |  |  |
|  | His |  |  | 0.40 |  |  |  |  |  |  |  |  |  |  |  |  |  |  |
|  | Ile |  |  |  |  |  |  | 0.42 |  |  |  |  |  |  |  |  |  |  |
|  | Leu |  |  |  |  |  |  |  | 0.82 |  |  |  |  |  |  |  |  |  |
|  | Lys |  |  | 0.53 |  |  |  |  |  |  |  |  |  |  |  |  |  |  |
|  | Met | 0.48 | 0.59 | 0.53 |  |  |  | 0.40 | 0.53 | 0.44 | 0.59 |  |  |  |  |  |  |  |
|  | Phe |  |  |  |  |  |  |  | 0.47 | 0.49 |  |  |  |  |  |  |  |  |
|  | Pro |  | 0.59 | 0.47 |  |  | 0.44 | 0.38 | 0.50 | 0.38 | 0.53 | 0.61 | 0.47 |  |  |  |  |  |
|  | Thr |  | 0.56 | 0.63 |  |  | 0.51 |  |  |  | 0.46 | 0.70 |  | 0.62 |  |  |  |  |
|  | Trp |  |  |  |  |  |  |  | 0.52 |  |  |  |  |  |  |  |  |  |
|  | Tyr | 0.44 | 0.43 |  |  |  |  |  | 0.68 | 0.56 | 0.46 | 0.62 | 0.41 | 0.58 |  |  |  |  |
|  | Val |  |  |  |  |  |  | 0.41 |  | 0.52 | 0.48 | 0.38 |  | 0.48 |  |  | 0.47 |  |
|  |  | Arg | Ala | Asn | Asp | Cys | Gln | His | Ile | Leu | Lys | Met | Phe | Pro | Thr | Trp | Tyr | Val |
|  |  | Plasma amino acid | | | | | | | | | | | | | | | | |

Correlation coefficients; *Spearman* (P<0.01).

**Table S5.** Correlation analysis – urinary protein glycation, oxidation and nitration free adducts.

| Urinary protein glycation, oxidation and nitration free adduct | Pyrraline |  |  |  |  |  |  |  |  |  |  |  |  |  |
| --- | --- | --- | --- | --- | --- | --- | --- | --- | --- | --- | --- | --- | --- | --- |
|  | FL |  |  |  |  |  |  |  |  |  |  |  |  |  |
|  | CML | 0.47 |  |  |  |  |  |  |  |  |  |  |  |  |
|  | G-H1 |  |  | 0.43 |  |  |  |  |  |  |  |  |  |  |
|  | MG-H1 | 0.42 | 0.38 |  |  |  |  |  |  |  |  |  |  |  |
|  | 3DG-H |  |  |  |  | 0.56 |  |  |  |  |  |  |  |  |
|  | CMA | 0.36 |  | 0.65 | 0.41 |  |  |  |  |  |  |  |  |  |
|  | GSP |  |  |  |  |  | 0.59 |  |  |  |  |  |  |  |
|  | DT | 0.46 |  | 0.55 |  | 0.45 |  | 0.50 |  |  |  |  |  |  |
|  | NFK |  |  |  |  |  | 0.47 |  | 0.56 |  |  |  |  |  |
|  | AASA |  |  | 0.42 |  |  |  | 0.44 |  | 0.44 | 0.48 |  |  |  |
|  | GSA | 0.40 | 0.37 | 0.44 |  |  |  | 0.56 |  | 0.53 |  | 0.83 |  |  |
|  | 3-NT |  |  |  |  | 0.34 | 0.40 |  |  |  |  |  |  |  |
|  |  | Pyrraline | FL | CML | G-H1 | MG-H1 | 3DG-H | CMA | GSP | DT | NFK | AASA | GSA | 3-NT |
|  |  | Urinary protein glycation, oxidation and nitration free adduct | | | | | | | | | | | | |

Correlation coefficients; *Spearman* (P<0.01).

**Table S6.** Correlation analysis – Urinary amino acids.

| Urinary amino acid | Arg |  |  |  |  |  |  |  |  |  |  |  |  |  |  |  |  |  |  |  |
| --- | --- | --- | --- | --- | --- | --- | --- | --- | --- | --- | --- | --- | --- | --- | --- | --- | --- | --- | --- | --- |
|  | Ala | 0.40 |  |  |  |  |  |  |  |  |  |  |  |  |  |  |  |  |  |  |
|  | Asn |  | 0.71 |  |  |  |  |  |  |  |  |  |  |  |  |  |  |  |  |  |
|  | Asp | 0.60 |  | 0.45 |  |  |  |  |  |  |  |  |  |  |  |  |  |  |  |  |
|  | Gln | 0.63 | 0.72 | 0.77 | 0.40 |  |  |  |  |  |  |  |  |  |  |  |  |  |  |  |
|  | Glu | 0.58 | 0.58 | 0.59 |  | 0.79 |  |  |  |  |  |  |  |  |  |  |  |  |  |  |
|  | Gly |  | 0.41 |  |  | 0.48 | 0.38 |  |  |  |  |  |  |  |  |  |  |  |  |  |
|  | His | 0.62 | 0.55 | 0.77 | 0.39 | 0.85 | 0.64 | 0.50 |  |  |  |  |  |  |  |  |  |  |  |  |
|  | Ile |  | 0.63 | 0.53 |  | 0.46 | 0.45 | 0.50 | 0.43 |  |  |  |  |  |  |  |  |  |  |  |
|  | Leu | 0.59 | 0.68 | 0.71 |  | 0.69 | 0.58 |  | 0.67 | 0.58 |  |  |  |  |  |  |  |  |  |  |
|  | Lys | 0.52 |  | 0.50 |  | 0.58 | 0.54 |  | 0.42 |  |  |  |  |  |  |  |  |  |  |  |
|  | Met | 0.50 | 0.75 | 0.76 |  | 0.74 | 0.57 |  | 0.65 | 0.43 | 0.52 | 0.57 |  |  |  |  |  |  |  |  |
|  | Phe | 0.50 | 0.66 | 0.54 |  | 0.64 | 0.55 |  | 0.65 | 0.54 | 0.76 | 0.47 | 0.63 |  |  |  |  |  |  |  |
|  | Pro | 0.47 | 0.61 | 0.58 |  | 0.61 | 0.42 |  | 0.42 | 0.53 | 0.69 | 0.53 | 0.62 | 0.40 |  |  |  |  |  |  |
|  | Ser | 0.68 | 0.71 | 0.84 | 0.44 | 0.92 | 0.69 | 0.49 | 0.84 | 0.42 | 0.66 | 0.40 | 0.76 | 0.55 | 0.63 |  |  |  |  |  |
|  | Thr | 0.57 | 0.75 | 0.71 | 0.37 | 0.85 | 0.62 | 0.41 | 0.73 | 0.43 | 0.72 | 0.56 | 0.91 | 0.63 | 0.62 | 0.84 |  |  |  |  |
|  | Trp | 0.48 | 0.66 | 0.54 |  | 0.68 | 0.64 |  | 0.60 | 0.55 | 0.72 | 0.39 | 0.64 | 0.71 | 0.45 | 0.51 | 0.65 |  |  |  |
|  | Tyr | 0.53 | 0.65 | 0.54 |  | 0.54 | 0.67 |  | 0.56 | 0.52 | 0.75 | 0.62 | 0.71 | 0.82 | 0.45 | 0.58 | 0.73 | 0.83 |  |  |
|  | Val | 0.47 | 0.60 | 0.51 | 0.42 | 0.48 | 0.43 |  | 0.50 | 0.65 | 0.75 | 0.43 | 0.67 | 0.64 | 0.52 | 0.48 | 0.69 | 0.77 | 0.70 |  |
|  |  | Arg | Ala | Asn | Asp | Gln | Glu | Gly | His | Ile | Leu | Lys | Met | Phe | Pro | Ser | Thr | Trp | Tyr | Val |

Correlation coefficients; *Spearman* (P<0.01).

**Table S7.**  Confusion matrix of algorithm to identify autistic spectrum disorder.

| Algorithm no | | 1 | | 2 | | 3 | | 4 | |
| --- | --- | --- | --- | --- | --- | --- | --- | --- | --- |
| nCorrect | | 12/14 | | 10/14 | | 14/14 | | 15/19 | |
|  | | Predicted class | | Predicted class | | Predicted class | | Predicted class | |
|  | | ASD | Control | ASD | Control | ASD | Control | ASD | Control |
| Clinical class | ASD | 12 | 2 | 10 | 4 | 14 | 0 | 15 | 4 |
|  | Control | 0 | 10 | 2 | 8 | 2 | 8 | 1 | 15 |

The confusion matrices above demonstrate representative results from one run of the classification experiment.
